# Exercise-mediated amelioration of high-fat diet-induced kidney injury: implications of microRNA regulation

**DOI:** 10.1080/0886022X.2025.2561221

**Published:** 2025-10-02

**Authors:** Yisheng Luan, Hang Tian, Jianrong Zheng, Yingzhe Xiong, Bing Zhang, Weihao Hong

**Affiliations:** a Division of Sports Science and Physical Education, Tsinghua University, Beijing, China; b Institute of Physical Education and Training, Capital University of Physical Education and Sports, Beijing, China; c School of Life Science and Technology, ShanghaiTech University, Shanghai, China; d School of Physical Education, Central China Normal University, Wuhan, China

**Keywords:** Exercise, treadmill, microRNA, obesity-related kidney injury, high-fat diet

## Abstract

Obesity-related kidney injury has become a critical health concern, driven by metabolic disturbances, chronic inflammation, oxidative stress, and structural remodeling. While exercise is widely recognized for its broad health benefits, including its ability to mitigate obesity-related complications by improving metabolic homeostasis, reducing inflammation, and enhancing antioxidative capacity, its specific role in protecting renal function – particularly through the modulation of microRNAs (miRNAs) – remains insufficiently understood. This study investigated the effects of an 8-week moderate-intensity treadmill exercise intervention on high-fat diet (HFD)-induced kidney injury in a murine model. Chronic HFD feeding resulted in significant renal dysfunction, evidenced by elevated filtration markers (urinary total protein, urinary microalbumin, urinary creatinine, and serum creatinine) and injury biomarkers (neutrophil gelatinase-associated lipocalin, cystatin C, and kidney injury molecule 1), alongside structural abnormalities, such as glomerular hypertrophy, mesangial expansion, and fibrosis. These changes were accompanied by increased pro-inflammatory cytokines (TNF-α, IL-6, and IL-1β), heightened oxidative stress (elevated malondialdehyde, reduced glutathione, glutathione s-transferase, and superoxide dismutase), and dysregulation of several miRNAs, such as miR-150-5p and miR-182-5p, which are known to exacerbate renal injury. Exercise significantly alleviated the adverse effects of HFD by improving renal function, reducing structural damage, suppressing inflammation, enhancing antioxidative capacity, and downregulating miR-150-5p and miR-182-5p. These findings underscore the therapeutic potential of exercise in mitigating obesity-related kidney injury and provide novel insights into its renoprotective effects mediated by miRNA regulation.

## Introduction

Obesity is a growing global health concern and a risk factor for several chronic diseases, including type 2 diabetes, cardiovascular conditions, and kidney disorders [1–3]. Among these, obesity-related kidney injury has gained increasing attention due to its complex pathogenesis and profound impact on renal function [4,5]. The progression of obesity-related kidney injury involves multiple interrelated mechanisms, such as hemodynamic alterations, lipotoxicity, chronic inflammation, and oxidative stress [6]. In particular, metabolic disturbances – including insulin resistance, dyslipidemia, and hyperuricemia – contribute to the activation of the renin-angiotensin-aldosterone system, exacerbating glomerular hyperfiltration, and promoting renal injury [7]. Mechanical compression from perirenal adipose tissue further impairs renal vascular dynamics, while adipose tissue dysfunction disrupts adipokine secretion patterns – elevating pro-inflammatory mediators (e.g., TNF-α and IL-6) and reducing protective ones (e.g., adiponectin and leptin) – thereby amplifying systemic inflammation and insulin resistance, which together accelerate kidney damage [8,9].

MicroRNAs (miRNAs) are a class of small, non-coding RNA molecules that play pivotal roles in regulating a wide array of biological processes, includ**i**ng inflammation, fibrosis, apoptosis, and oxidative stress – key factors in the onset and progression of renal pathologies, such as acute kidney injury (AKI), chronic kidney disease (CKD), and diabetic nephropathy (DN) [10,11]. For instance, miR-21 is implicated in promoting fibrotic and inflammatory responses by activating critical signaling pathways, including TGF-β and NF-κB, thus contributing to kidney damage and disease progression [12]. MiR-155 is involved in kidney injury and fibrosis, where it regulates autophagy and fibrosis through the SOCS1/SOCS6/STAT3 pathway, particularly under high glucose conditions in diabetic kidney disease [13,14]. In contrast, miR-126 plays a protective role in renal damage by modulating inflammatory and fibrotic pathways; elevated levels of miR-126 are associated with improved outcomes in diabetic kidney disease and CKD [15,16].

Recent studies have shown that physical exercise can modulate miRNA expression across various tissues, providing a potential molecular link between exercise and its protective effects against obesity-related dysfunction [17–19]. In skeletal muscle, exercise upregulates miR-1, miR-133a, and miR-206 to promote myogenesis and regeneration, while miR-486 enhances glucose uptake *via* the PI3K/AKT signaling cascade [20,21]. In the liver, exercise downregulates miR-122, which is associated with lipid accumulation, and miR-34a, a negative regulator of the SIRT1 pathway, thereby improving hepatic insulin sensitivity [22]. In adipose tissue, miR-27a is suppressed by exercise to enhance lipolysis and promote browning, while other miRNAs, such as miR-378 and miR-143 are involved in adipogenesis and inflammation modulation [23]. Additionally, miRNAs such as miR-221 and miR-222 may act as circulating mediators of exercise-induced inter-organ communication, coordinating metabolic responses across tissues [24]. Despite these advances, the effects of exercise on miRNA expression in the kidney – particularly under conditions of obesity-induced injury – remain poorly understood. Given the central role of miRNAs in renal pathology, understanding how exercise influences renal miRNA profiles may offer new insights into the mechanisms of exercise-induced renal protection.

To address this gap, this study examines the therapeutic effects of an 8-week moderate-intensity treadmill exercise regimen in a mouse model of high-fat diet (HFD)-induced obesity-related kidney injury and profiles renal miRNA expression to investigate the potential role of miRNAs in exercise-mediated renoprotection. The results provide preliminary evidence that miRNAs may contribute to the beneficial effects of exercise on obesity-related kidney injury and underscore the potential of exercise as a non-pharmacological strategy for its management.

## Materials and methods

### Animals

Three-week-old male C57BL/6 mice (*n* = 48) were maintained at a temperature of 22 ± 2 °C and a humidity of 50% ± 5%, under a 12-h light-dark cycle. Following a one-week acclimation period, mice were randomly assigned to four groups (each *n* = 12): standard chow diet (C), standard chow diet with exercise (CE), high-fat diet (H), and high-fat diet with exercise (HE). The standard chow diet (D12450J, with 10 kcal% fat) and HFD (D12492, with 60 kcal% fat) were purchased from Research Diets. This study was approved by the Institutional Animal Care and Use Committee of Tsinghua University (Approval No: THU-LARC-2023-004) and was conducted in accordance with the ARRIVE guidelines.

### Exercise protocol

**Figure 1.**
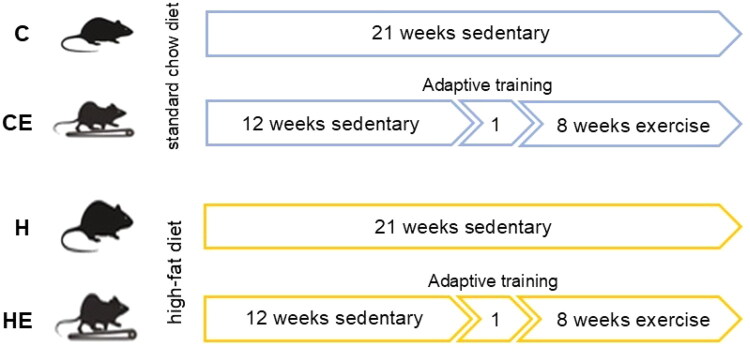
Schematic overview of the training and dietary regimens.

A schematic overview of the training and dietary interventions is shown in Figure 1. After 12 weeks of feeding, exercise was conducted using an animal treadmill at an intensity corresponding to 60% VO_2_ max, as previously described [25]. Briefly, mice in the exercise groups underwent an adaptive training phase at a speed of 7 m/min for one week, with sessions lasting 10 min per day. This was followed by an 8-week formal training regimen, performed 5 d per week for 45 min per session, starting with a 10-min warm-up at 10 m/min. The training pace was progressively increased: 13 m/min in weeks 1–2, 15 m/min in weeks 3–4, and 17 m/min in weeks 5–8.

### Body weight, blood, and urine parameters measurements

Body weight was monitored and recorded weekly. Urine was collected in metabolic cages overnight after the last training session. The following day, mice were anesthetized with Avertin (intraperitoneally), and blood was collected *via* retro-orbital bleeding under deep anesthesia. Mice were then euthanized by cervical dislocation, and kidneys were immediately harvested. Serum was isolated by centrifugation at 3000 rpm for 15 min at 4 °C. Urinary total protein (TP), microalbumin (mALB), and creatinine levels were quantified using Urine Protein Content Assay Kit, Mouse Microalbuminuria ELISA Kit, and Creatinine Assay kit (Sangon Biotech, Shanghai, China), respectively. Serum creatinine (Scr), neutrophil gelatinase-associated lipocalin (NGAL), and cystatin C (Cys-C) levels were measured using Creatinine Assay kit (Sangon Biotech), Mouse NGAL/Lipocalin-2 ELISA Kit, and Mouse Cys-C ELISA Kit (Beyotime, Shanghai, China), respectively. All assays were performed in accordance with the manufacturers’ instructions.

### Kidney histology and morphometric analysis

Kidneys were fixed in 4% paraformaldehyde and embedded in paraffin. Tissue sections (4 μm) were then stained with periodic acid Schiff (PAS), Masson’s trichrome, and TUNEL staining according to the manufacturer’s instructions (Solarbio Life Sciences, Beijing, China). The relative glomerular size, proportion of fibrotic area, and proportion of apoptotic cells were analyzed using ImageJ software, with 10 non-overlapping areas selected for each sample.

### Oxidative stress measurement

Oxidative stress levels in kidney tissues were assessed using Glutathione S-transferase (GST) Activity Assay Kit, Glutathione (GSH) Content Assay Kit, Superoxide dismutase (SOD) Activity Assay Kit, and Malondialdehyde (MDA) Content Assay Kit. All assays were performed according to the manufacturer’s instructions (Solarbio Life Sciences).

### Quantitative real-time PCR

Kidney total RNA extraction was performed with Trizol (Invitrogen, Carlsbad, CA) according to the manufacturer’ s instructions. cDNA was synthesized using HiScript II Q RT SuperMix (Vazyme, Nanjing, China). Quantitative RT-PCR was performed using AceQ qPCR SYBR Green Master Mix (Vazyme).

The following primers were used:

*Gapdh     *Forward: AGAAGGTGGTGAAGCAGGCATCT
 *         *Reverse: CGGCATCGAAGGTGGAAGAGTG
*Kim-1      *Forward: GCGTGTCACCTATCAGAAGAGCAGTC
 *         *Reverse: CCAGGAATCTCCACTCGACAACAAT
*Il6          *Forward: TAGTCCTTCCTACCCCAATTTCC
 *           *Reverse: TTGGTCCTTAGCCACTCCTTC
*Il1b      *Forward: GCAACTGTTCCTGAACTCAACT
 *            *Reverse: ATCTTTTGGGGTCCGTCAACT
*Tnfa           *Forward: CCCTCACACTCAGATCATCTTCT
 *            *Reverse: GCTACGACGTGGGCTACAG
U6        * *Forward: CGCTTCGGCAGCACATATACTAA
           *  *Reverse: GCTGTCAACGATACGCTACCTA
miR-182-5p      * *Forward: GGCAATGGTAGAACTCACA
 *            *Reverse: GAACATGTCTGCGTATCTC
miR-150-5p * *Forward: CATGGCCCTGTCTCCCAAC
 *      *Reverse: GGCCTGTACCAGGGTCTGA

### MiRNA sequencing

MiRNA sequencing was performed on kidney tissue samples using the Illumina HiSeq platform by Biomarker Technologies (Beijing, China). Raw sequencing reads underwent quality control to remove adapter sequences, low-quality reads, and reads outside the 18–30 nucleotide range, yielding high-quality clean reads. Clean reads were aligned to the Mus_musculus. GRCm38 reference genome using Bowtie, and annotated miRNAs were identified by mapping to miRBase version 22 (Manchester, United Kingdom). Expression levels were quantified using the transcripts per million (TPM) normalization approach, and differential expression analysis was conducted using edgeR with criteria of |fold change| ≥ 1.5 and *p* value ≤ 0.05, with FDR correction for multiple testing.

### Statistical analysis

Statistical analyses were performed using GraphPad Prism version 7 (GraphPad Software, Inc, La Jolla, CA). Data are expressed as mean ± standard deviation. One-way analysis of variance (ANOVA) was employed for comparisons among multiple groups, followed by Tukey’ s post-hoc test for pairwise comparisons. A *p* value of < 0.05 was considered statistically significant.

## Results

### The protective effects of exercise against HFD-induced renal dysfunction

**Figure 2.**
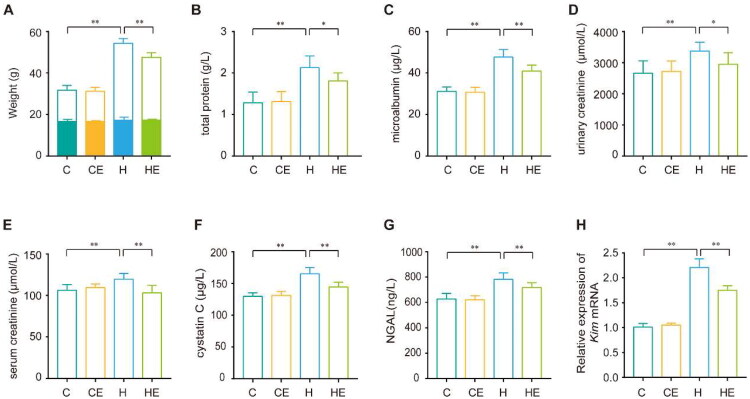
The protective effects of exercise against HFD-induced renal dysfunction. (A) body weight in the last week, the colored portion of each bar represents the initial body weight. (B–D) total protein (TP), microalbumin (mALB), and creatinine (ucr) levels in the urine of the C, CE, H, and HE groups. (E–G) creatinine (scr), cystatin C (Cys-C), and neutrophil gelatinase-associated lipocalin (NGAL) levels in the serum of the C, CE, H, and HE groups. (H) Expression of kidney injury molecule 1 (*kim-1*) mRNA in the kidneys of the C, CE, H, and HE groups, with results shown as relative expression normalized to *gapdh* mRNA levels. Each group *n* = 12. **p < 0.01; *p < 0.05.

After 21 weeks of HFD feeding, the body weight of the H group was significantly higher than that of the C group, reflecting the impact of prolonged HFD consumption. Notably, exercise intervention resulted in a reduction in body weight in the HE group compared to the H group, suggesting its potential to counteract weight gain induced by HFD (Figure 2(A)).

HFD significantly elevated markers of renal filtration function, including urinary TP, mALB, urinary creatinine (Ucr), and serum creatinine (Scr), which increased by 67.7%, 53%, 26.5%, and 12.6%, respectively, in the H group compared to the C group. These findings indicate impaired renal filtration capacity caused by HFD. Exercise intervention in the HE group significantly improved these parameters, reducing TP, mALB, Ucr, and Scr levels by 15.1%, 14.3%, 12.6%, and 13.9%, respectively, compared to the H group (Figure 2(B–E)).

Additionally, renal injury biomarkers such as Cys-C, NGAL, and kidney injury molecule 1 (*Kim-1*) mRNA were markedly upregulated in the H group, showing increases of 27.4%, 24.6%, and 132.6%, respectively, relative to the C group. Exercise intervention in the HE group significantly lowered these biomarkers, reducing Cys-C, NGAL, and *Kim-1* mRNA levels by 12.8%, 8.2%, and 30.7%, respectively (Figure 2(F–H)). These results underscore the protective effects of exercise in improving renal filtration function and reducing markers of renal injury.

### Exercise mitigates HFD-induced renal fibrosis, apoptosis, and structural alterations

**Figure 3.**
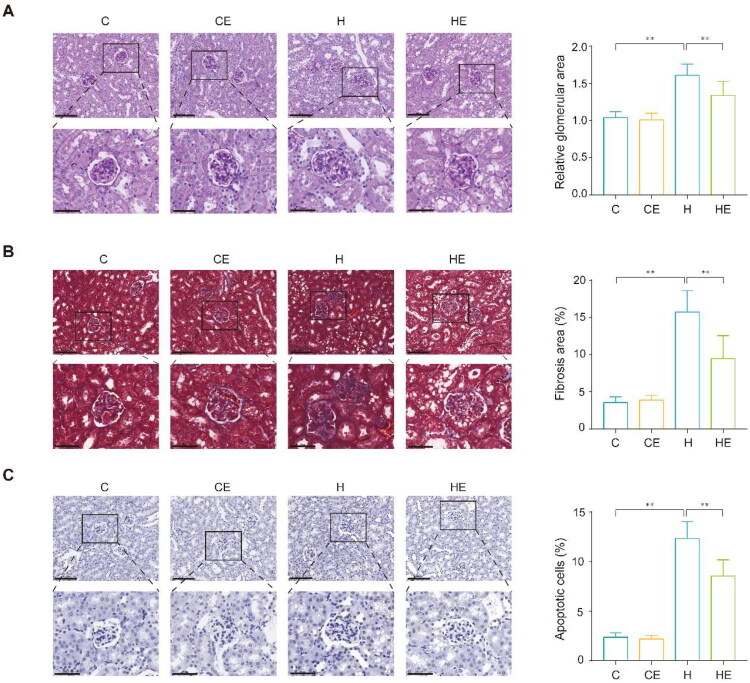
Exercise mitigates HFD-induced renal fibrosis, apoptosis, and structural alterations. (A) PAS staining of kidney sections from the C, CE, H, and HE groups (scale bars: 100 µm, top; 50 µm, bottom), with quantified relative glomerular size shown on the right. (B) Masson staining of kidney sections (scale bars: 100 µm, top; 50 µm, and bottom), with fibrotic area proportion quantified on the right. (C) TUNEL staining of kidney sections (scale bars: 100 µm, top; 50 µm, bottom), with apoptotic cell proportion shown on the right. Each group *n* = 12. **p < 0.01.

HFD induced significant structural abnormalities in the kidneys, as evidenced by PAS staining. Observations included hypertrophic glomeruli, dilated Bowman’s capsules, thickened glomerular capillaries, and an expanded mesangial matrix, culminating in a 54.8% increase in glomerular area compared to the C group. Exercise intervention in the HE group partially alleviated these changes, reducing glomerular area by 16.8% (Figure 3(A)).

Masson staining further revealed excessive collagen deposition in the glomerular and interstitial regions of the H group, indicative of advanced renal fibrosis. The fibrotic area increased by 339.6% relative to the C group, reflecting substantial extracellular matrix accumulation. Exercise intervention significantly mitigated this effect, reducing the fibrotic area by 40%, thereby decelerating fibrosis progression (Figure 3(B)).

TUNEL staining demonstrated marked apoptotic activity in the H group, with a 425.1% increase in apoptotic cell ratio compared to the C group. This extensive cell death contributed to the structural and functional deterioration of the kidneys under HFD conditions. Exercise intervention reduced apoptosis by 30.8%, underscoring its protective role in preserving renal tissue integrity and limiting HFD-induced cellular damage (Figure 3(C)).

### Exercise alleviates HFD-induced renal oxidative stress and inflammation

**Figure 4.**
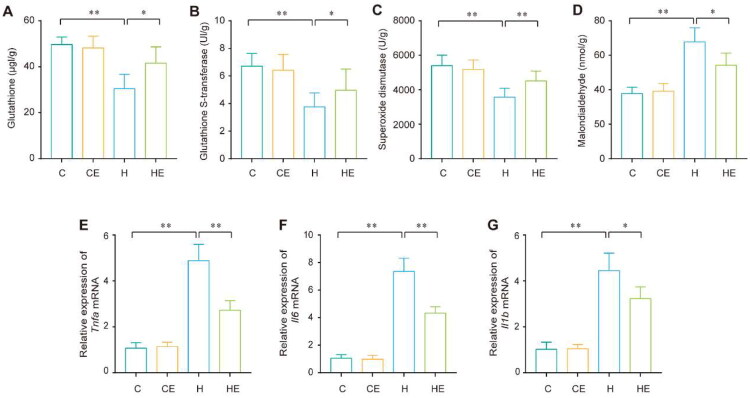
Exercise alleviates HFD-induced renal inflammation and oxidative stress. (A–D) glutathione (GSH), glutathione S-transferase (GST), superoxide dismutase (SOD), and malondialdehyde (MDA) levels in the kidneys of the C, CE, H, and HE groups. (E–G) tumor necrosis factor alpha (*tnfa*), interleukin 6 (*Il6*), and interleukin 1 beta (*Il1b)* mRNA expression in the kidneys of the C, CE, H, and HE groups, with results shown as relative expression normalized to *gapdh* mRNA levels. Each group *n* = 12. **p < 0.01; *p < 0.05.

In the H group, oxidative stress was evidenced by a significant reduction in the levels of antioxidant markers, including GSH, GST, and SOD, which decreased by 38.8%, 45.5%, and 33.9%, respectively. Concurrently, MDA, a marker of lipid peroxidation, exhibited a 79% increase compared to the C group. In contrast, exercise intervention in the HE group effectively mitigated HFD-induced oxidative stress, as demonstrated by a restoration of antioxidant capacity. Specifically, GSH, GST, and SOD levels increased by 36.5%, 35.5%, and 26.4%, respectively, while MDA levels decreased by 20% (Figure 4(A–D)).

Furthermore, the H group exhibited a marked elevation in pro-inflammatory cytokine expression, including TNF-α, IL-6, and IL-1β, with increases of 356%, 600%, and 333.3%, respectively, compared to the C group. In contrast, the HE group showed substantial reductions in these inflammatory markers, with decreases of 44.3%, 41.2%, and 27%, respectively, following exercise intervention (Figure 4(E–G)). These findings confirm the antioxidative and anti-inflammatory effects of exercise in ameliorating HFD-induced renal oxidative stress and inflammation.

### Exercise-mediated regulation of renal miRNAs in HFD condition

**Figure 5.**
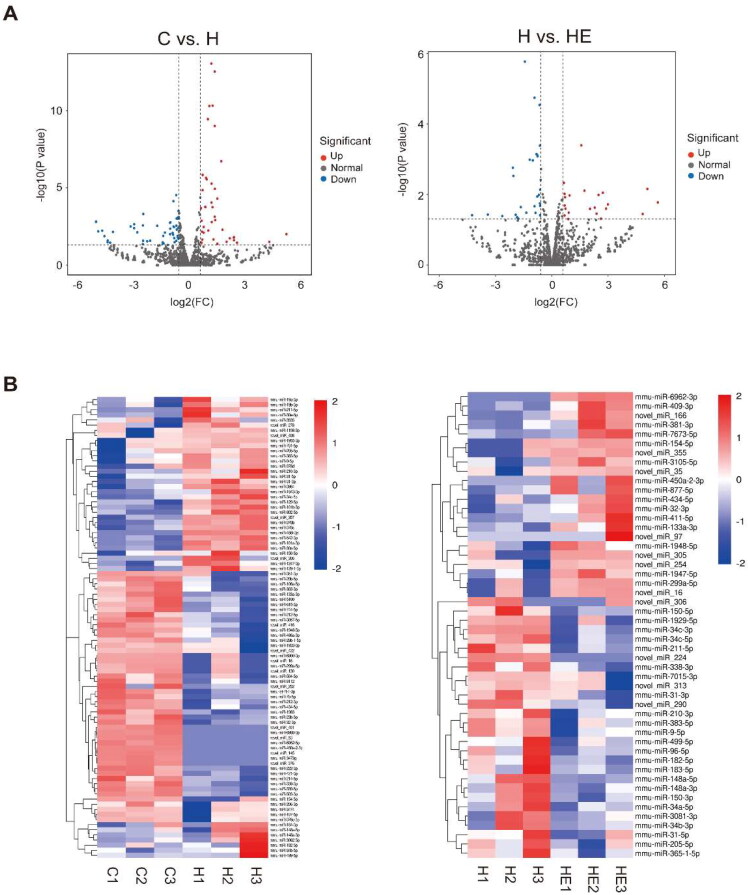
Profiling of differentially expressed renal miRNAs in response to HFD and exercise. (A) Volcano plots show differentially expressed miRNAs in C *vs*. H and H *vs.* HE. (B) Hierarchical clustering analysis of differentially expressed miRNAs in C *vs*. H and H *vs*. HE.

**Figure 6.**
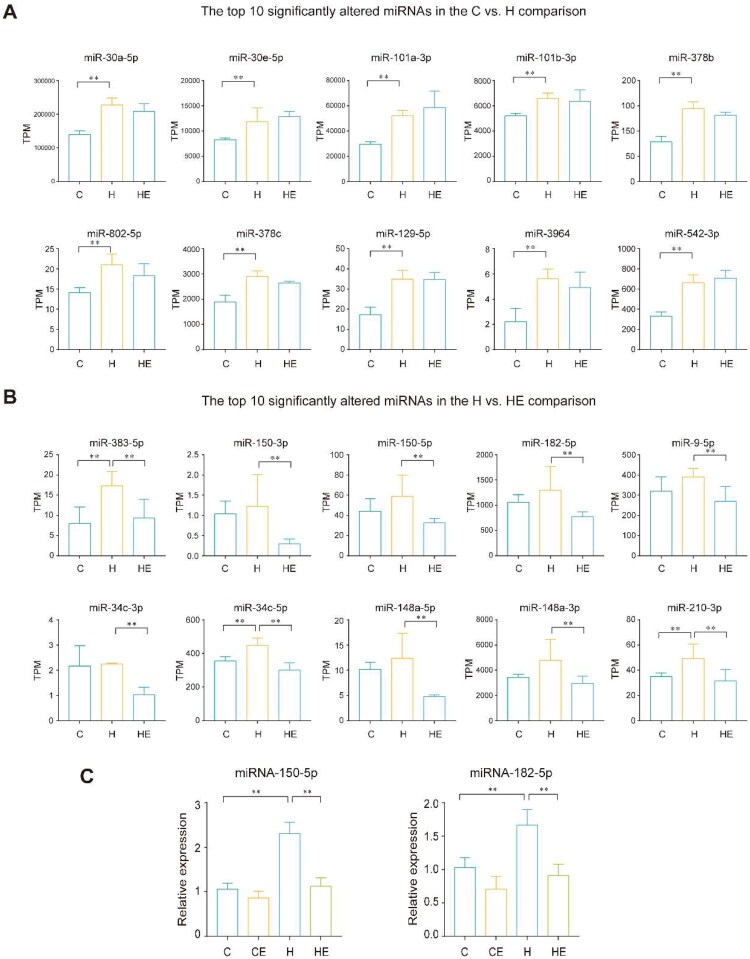
Exercise-mediated modulation of renal miRNAs in HFD condition. (A) The top 10 significantly altered miRNAs in the C *vs.* H comparison. (B) The top 10 significantly altered miRNAs in the H *vs*. HE comparison. (C) The expression levels of miRNA-150-5p and miRNA-182-5p in the kidneys of the C, CE, H, and HE groups were validated by qPCR, with results shown as relative expression normalized to U6. miRNA-seq each group *n* = 3. qPCR each group *n* = 12. **p < 0.01.

To explore the molecular mechanisms underlying these effects, we analyzed kidney miRNA profiling in the C, H, HE groups. Differentially expressed miRNAs were screened with criteria of |fold change| ≥ 1.5 and *p* value ≤ 0.05. In the C *vs*. H comparison, a total of 90 differentially expressed miRNAs were identified, including 41 upregulated and 49 downregulated miRNAs. In the H *vs*. HE comparison, a total of 50 differentially expressed miRNAs were identified, with 22 upregulated and 28 downregulated miRNAs (Figure 5(A,B)). The top 10 significantly altered miRNAs in the C *vs.* H comparison included miR-101a-3p, miR-542-3p, miR-378b, miR-30a-5p, miR-378c, miR-129-5p, miR-3964, miR-101b-3p, miR-30e-5p, and miR-802-5p (Figure 6(A), S1 Table). In the H *vs.* HE comparison, the top 10 significantly altered miRNAs included miR-148a-5p, miR-150-5p, miR-34c-5p, miR-9-5p, miR-182-5p, miR-148a-3p, miR-210-3p, miR-34c-3p, miR-383-5p, and miR-150-3p (Figure 6(B), S2 Table).

Among these, miR-150-5p and miR-182-5p, previously identified as promoters of kidney disease progression, exhibited significant upregulation in the H group and notable downregulation in the HE group following exercise intervention, a pattern further validated by qPCR (Figure 6(C)). These findings suggest that prolonged HFD exposure induces elevated levels of miR-150-5p and miR-182-5p, potentially contributing to kidney damage, while exercise may alleviate HFD-induced renal injury by downregulating these miRNAs.

## Discussion

Chronic exposure to HFD is a well-established driver of metabolic disturbances and kidney injury, with its pathogenesis involving complex interactions among impaired filtration, structural remodeling, and molecular dysregulation [26]. Although exercise is widely recognized for its systemic benefits, including its ability to influence miRNA expression, its specific role in modulating miRNA-mediated mechanisms in HFD-induced kidney injury remains insufficiently understood. This study provides novel insights into the multifaceted protective effects of exercise, demonstrating its capacity to restore renal function, preserve structural integrity, and regulate miRNA expression to mitigate HFD-induced kidney damage.

Consistent with previous studies, prolonged HFD feeding led to significant renal damage, characterized by impaired filtration function and structural abnormalities [7,27,28]. Functional biomarkers, including TP, mALB, Ucr, and Scr, were significantly elevated. Additionally, injury markers such as Cys-C, NGAL, and *Kim-1* mRNA levels were increased, indicating both diminished renal filtration capacity and exacerbated tissue damage. These findings underscored the association between metabolic overload and renal dysfunction, highlighting the adverse effects of HFD on kidney health. Exercise intervention reversed these trends, significantly improving these markers and demonstrating its ability to restore filtration function and mitigate renal injury. These functional benefits were accompanied by structural improvements, as exercise alleviated HFD-induced glomerular hypertrophy, mesangial expansion, and excessive collagen deposition, all hallmarks of advanced fibrosis. By reducing glomerular size, fibrosis, and apoptosis, exercise preserved renal architecture and slowed pathological remodeling, underscoring its broad protective effects.

The structural and functional benefits observed could be partly attributed to exercise’s anti-inflammatory and antioxidative roles. HFD exposure significantly elevated pro-inflammatory cytokines (TNF-α, IL-6, and IL-1β) and oxidative stress markers (reduced GSH, GST, SOD; increased MDA), both of which exacerbate renal damage. Exercise intervention effectively suppressed these inflammatory mediators and restored antioxidant defenses, demonstrating its systemic role as a modulator of immune and oxidative pathways. This interplay between inflammation, oxidative stress, and structural damage highlights the interconnected mechanisms underlying HFD-induced kidney injury and the multifactorial benefits of exercise.

At the molecular level, notable changes in miRNA expression were observed under HFD and exercise intervention, with 90 differentially expressed miRNAs identified in the C *vs*. H group (41 upregulated and 49 downregulated) and 50 in the H *vs*. HE group (22 upregulated and 28 downregulated). Among the C *vs*. H group, the top 10 significantly differentially expressed miRNAs included miR-101a-3p, miR-542-3p, miR-378b, miR-30a-5p, miR-378c, miR-129-5p, miR-3964, miR-101b-3p, miR-30e-5p, and miR-802-5p. MiR-542-3p and miR-30e-5p stand out for their established roles in promoting fibrosis and apoptosis in kidney disease. Li et al. demonstrated that miR-542-3p was significantly upregulated in a rat model of renal tubular interstitial fibrosis induced by unilateral ureteral obstruction. Functional studies revealed that miR-542-3p promotes fibrosis by suppressing AGO1 expression *via* binding to its 3′UTR, driving a fibrotic phenotype in proximal tubular epithelial cells [29]. Similarly, Liu et al. identified elevated miR-30e-5p levels in HK-2 cells treated with Urografin, a model for AKI. MiR-30e-5p suppressed Beclin1 expression, impairing autophagy, and heightened apoptosis through increased caspase 3 expression, further underscoring its pathogenic role in renal injury [30].

In the H *vs*. HE comparison, the top 10 significantly differentially expressed miRNAs included miR-148a-5p, miR-150-5p, miR-34c-5p, miR-166, miR-9-5p, miR-182-5p, miR-148a-3p, miR-210-3p, miR-34c-3p, and miR-383-5p. Among these, miR-150-5p and miR-182-5p have been reported to be implicated in kidney disease progression. Zhou et al. showed that hypoxia-induced exosomes enriched in miR-150-5p activated renal fibroblasts and exacerbated fibrosis by suppressing cytokine signaling inhibitor 1 expression. Inhibition of miR-150-5p-containing exosomes attenuated fibrosis both *in vitro* and *in vivo*, validating its regulatory role in fibrotic processes [31]. On the other hand, Ding et al. reported that miR-182-5p was significantly upregulated in ischemia-reperfusion kidney injury and suppressed the ferroptosis-resistance genes GPX4 and SLC7A11. This suppression exacerbated oxidative damage, promoting ferroptosis, and aggravating renal pathology. Notably, silencing miR-182-5p alleviated kidney damage, emphasizing its importance in renal injury progression [32]. The significant upregulation of miR-150-5p and miR-182-5p in the H group, combined with their pronounced downregulation following exercise intervention in the HE group, indicates that prolonged HFD consumption induced aberrant expression of these miRNAs, potentially contributing to renal pathology. Conversely, exercise appeared to attenuate this effect by restoring their expression to baseline levels, emphasizing their potential role in the mechanisms underlying exercise-mediated renal protection.

Despite these encouraging findings, several limitations of this study should be acknowledged. First, all experiments were conducted using male mice, which may limit the generalizability of the results. Given that sex hormones influence both renal physiology and response to metabolic stress, future studies should include both sexes to account for potential sex-specific differences. Second, although the duration of HFD feeding and exercise intervention was sufficient to elicit clear phenotypic and molecular changes, a longer observation period may be required to assess the long-term efficacy and durability of exercise-induced renal protection. Third, while this study identified several differentially expressed miRNAs potentially involved in renal injury and its mitigation by exercise, their specific functions and target genes were not experimentally validated. Functional assays, such as overexpression or inhibition of candidate miRNAs, are still needed to elucidate their mechanistic roles. Finally, this study relied solely on miRNA sequencing to explore molecular changes. Integrating multi-omics strategies in future investigations will help to construct a more comprehensive molecular landscape and clarify the interplay between miRNAs and other regulatory layers.

## Conclusion

This study highlights the protective role of exercise in mitigating HFD-induced kidney injury, as it restores renal function, preserves structural integrity, alleviates inflammation and oxidative stress, and downregulates renal injury-promoting miRNAs such as miR-150-5p and miR-182-5p. These findings offer valuable insights into the mechanisms by which exercise confers renal protection and highlight its therapeutic potential in mitigating obesity-related kidney diseases.

## Supplementary Material

MASSON HE group.jpg

TUNEL H group.jpg

MASSON CE group.jpg

S1 Table.xlsx

TUNEL C group.jpg

TUNEL CE group.jpg

MASSON H group.jpg

PAS CE group.jpg

S2 Table.xlsx

PAS HE group.jpg

MASSON C group.jpg

TUNEL HE group.jpg

PAS H group.jpg

PAS C group.jpg
