# Supplementary figures and images for: Exercise-mediated amelioration of high-fat diet-induced kidney injury: implications of microRNA regulation
Source: Ren Fail. 2025 Oct 2;47(1):2561221. doi: 10.1080/0886022X.2025.2561221 (PMC12498360; doi:10.1080/0886022X.2025.2561221)

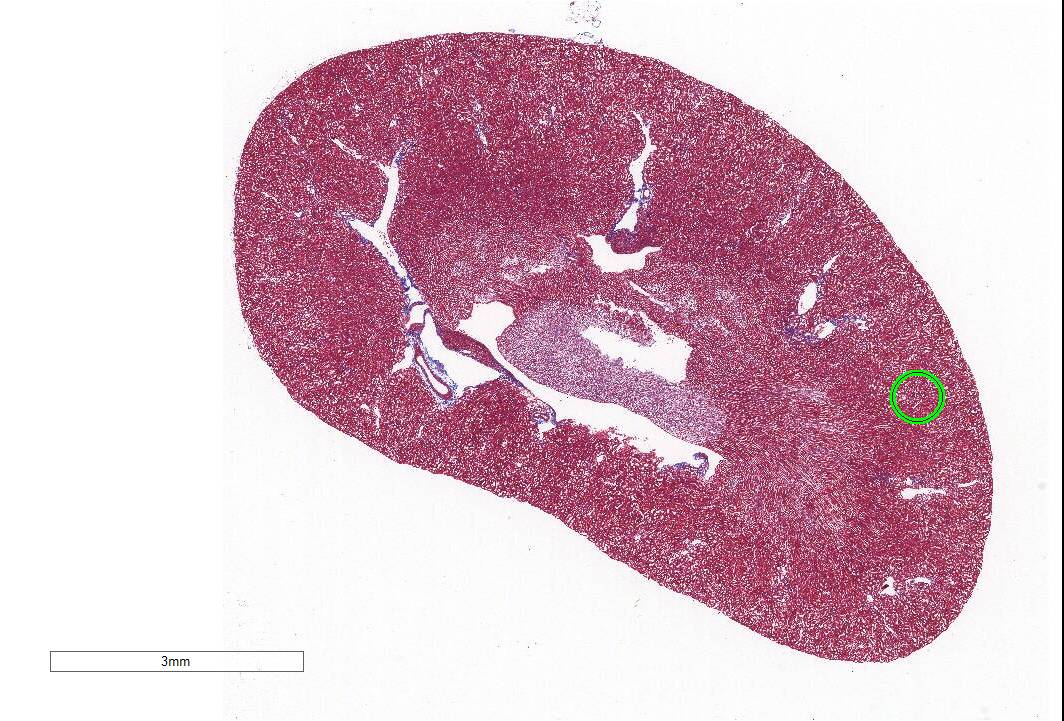

Supplement: MASSON HE group.jpg [file IRNF_A_2561221_SM0250.jpg]

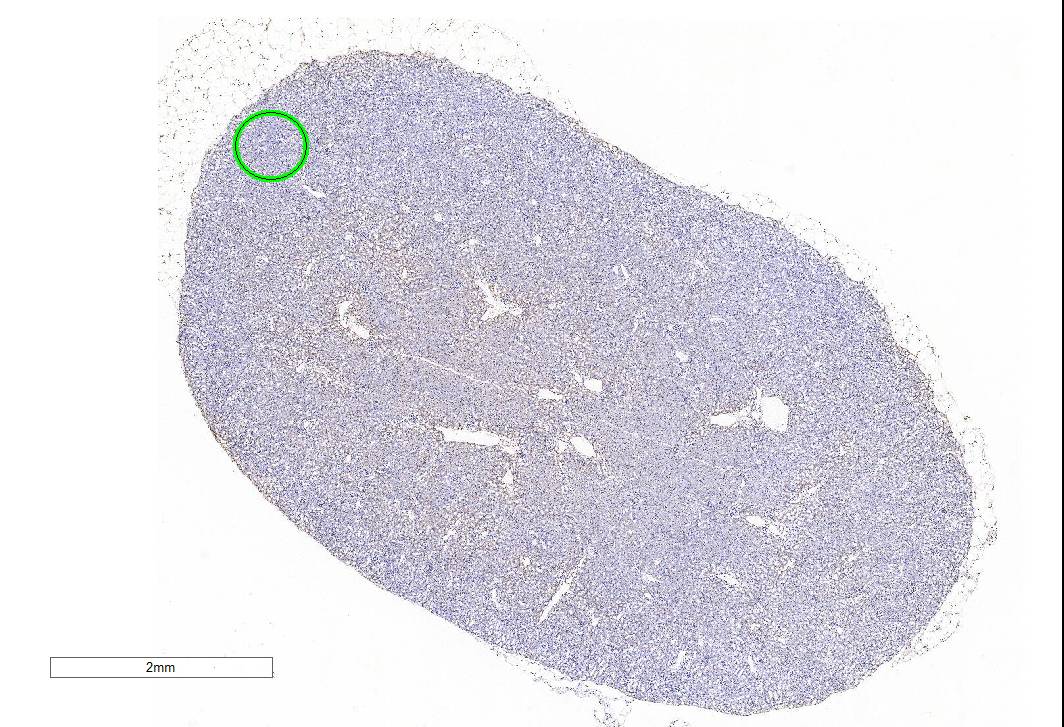

Supplement: TUNEL H group.jpg [file IRNF_A_2561221_SM0249.jpg]

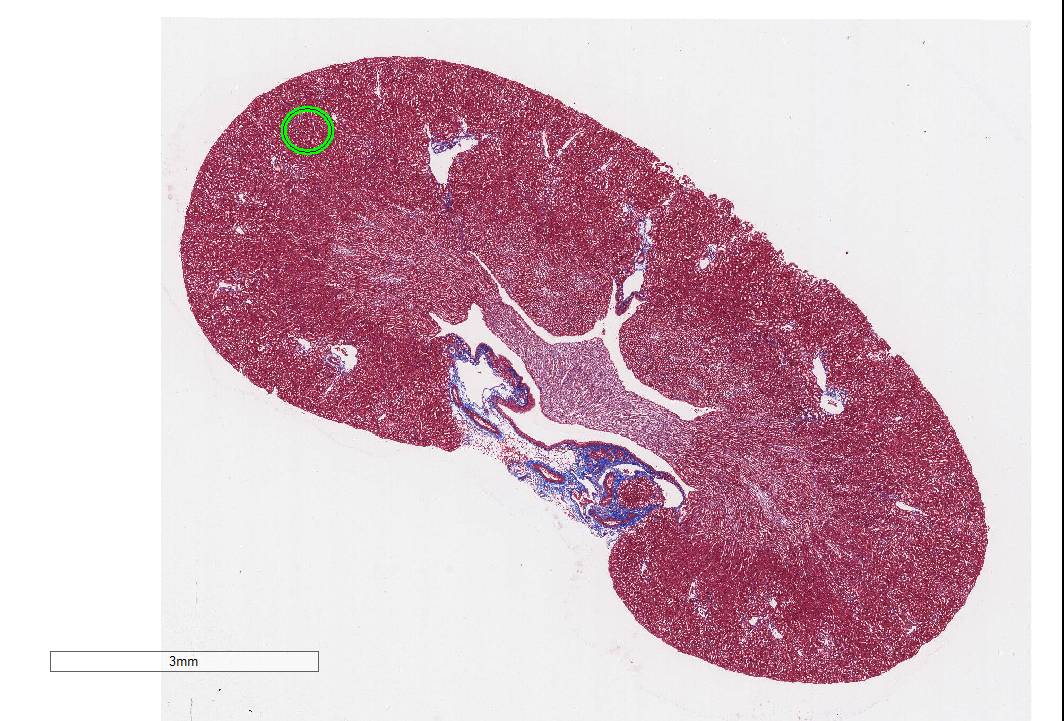

Supplement: MASSON CE group.jpg [file IRNF_A_2561221_SM0248.jpg]

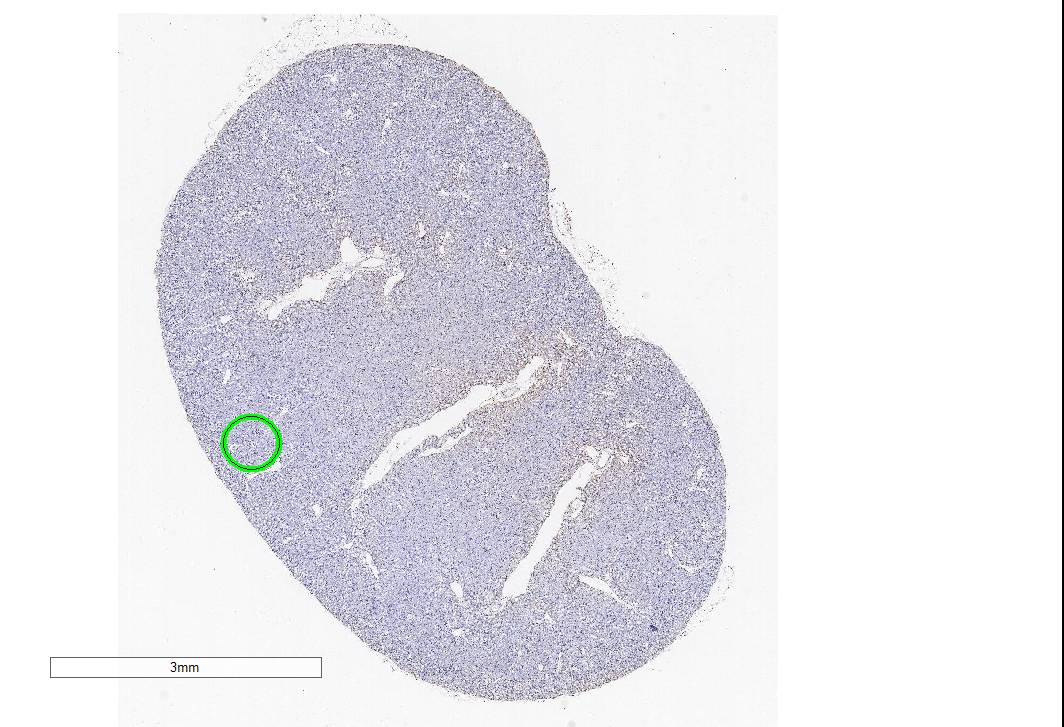

Supplement: TUNEL C group.jpg [file IRNF_A_2561221_SM0246.jpg]

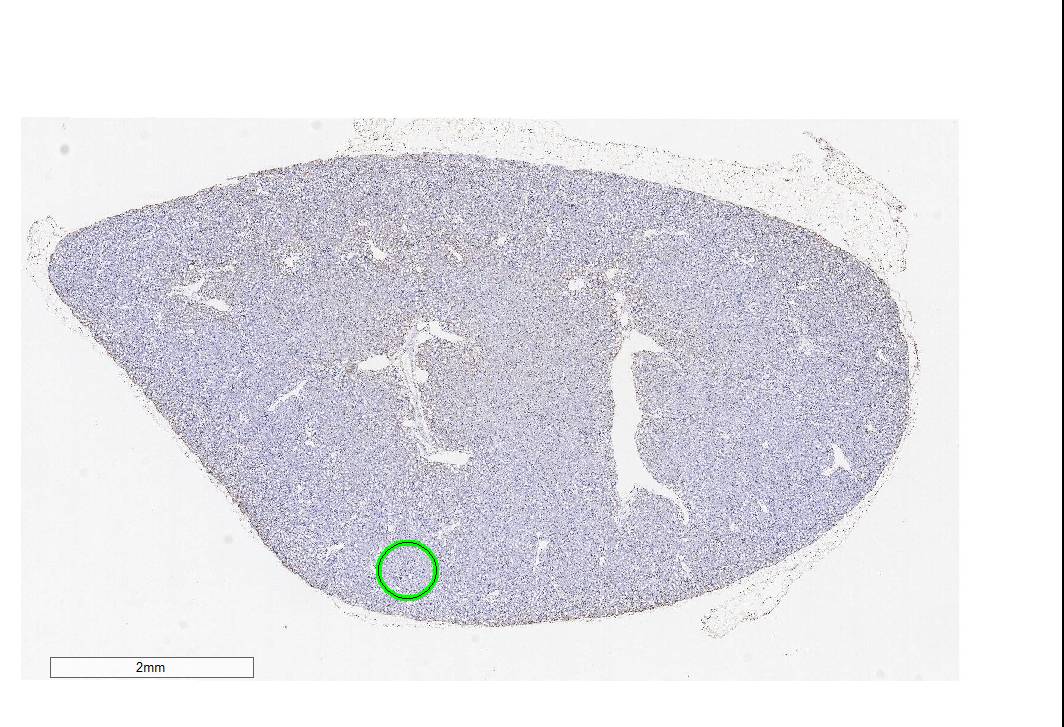

Supplement: TUNEL CE group.jpg [file IRNF_A_2561221_SM0245.jpg]

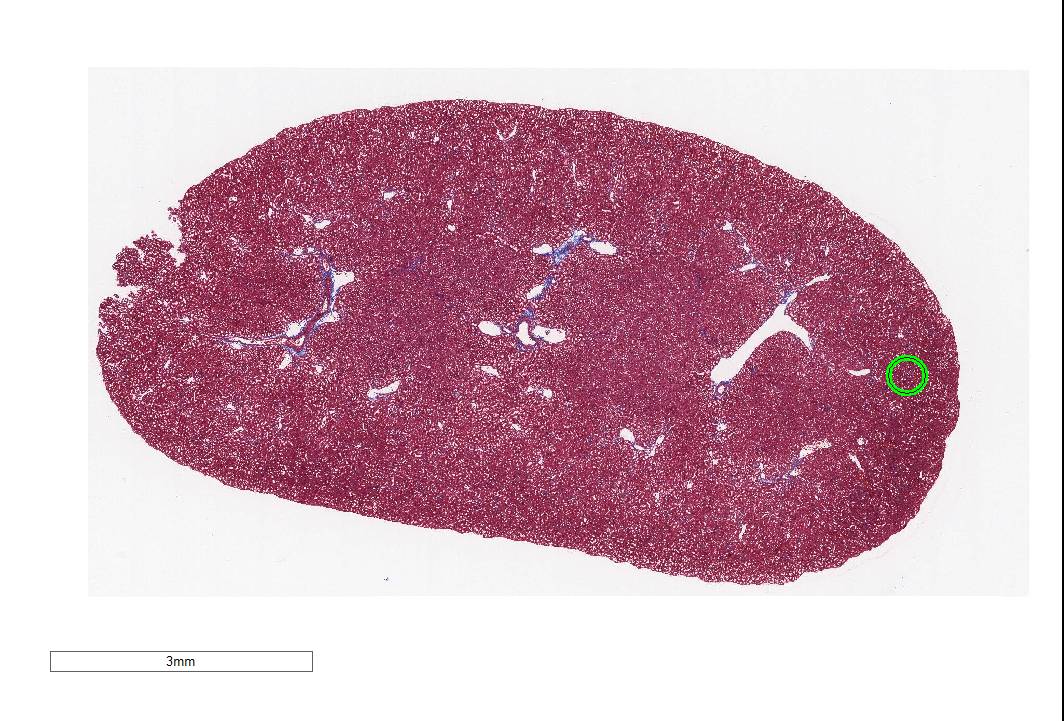

Supplement: MASSON H group.jpg [file IRNF_A_2561221_SM0244.jpg]

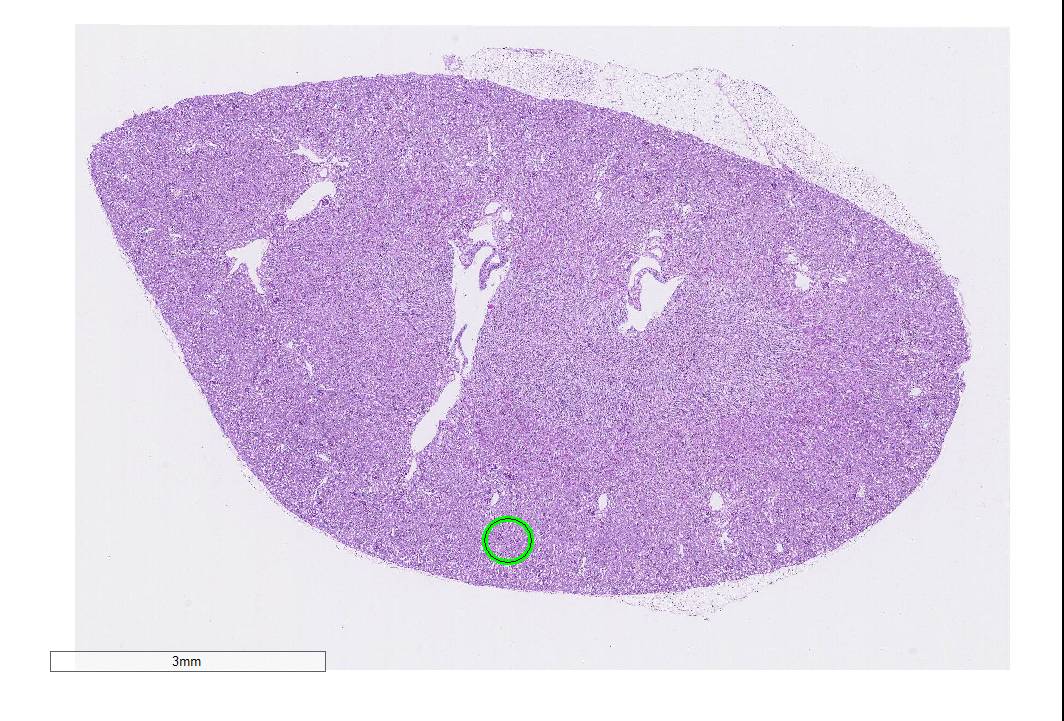

Supplement: PAS CE group.jpg [file IRNF_A_2561221_SM0243.jpg]

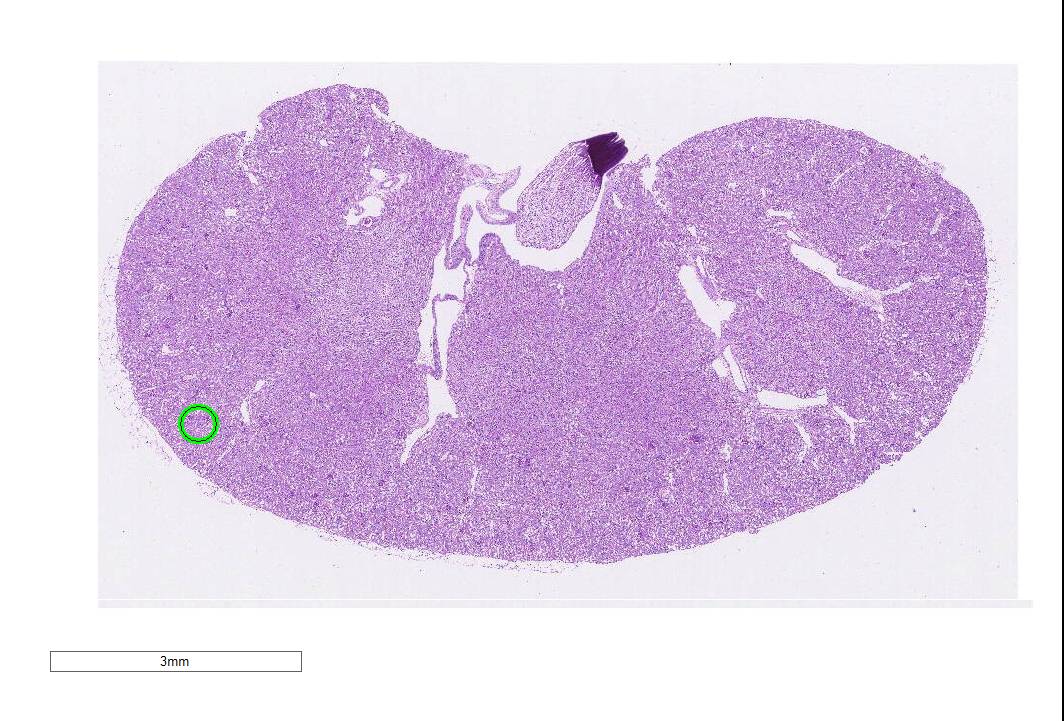

Supplement: PAS HE group.jpg [file IRNF_A_2561221_SM0241.jpg]

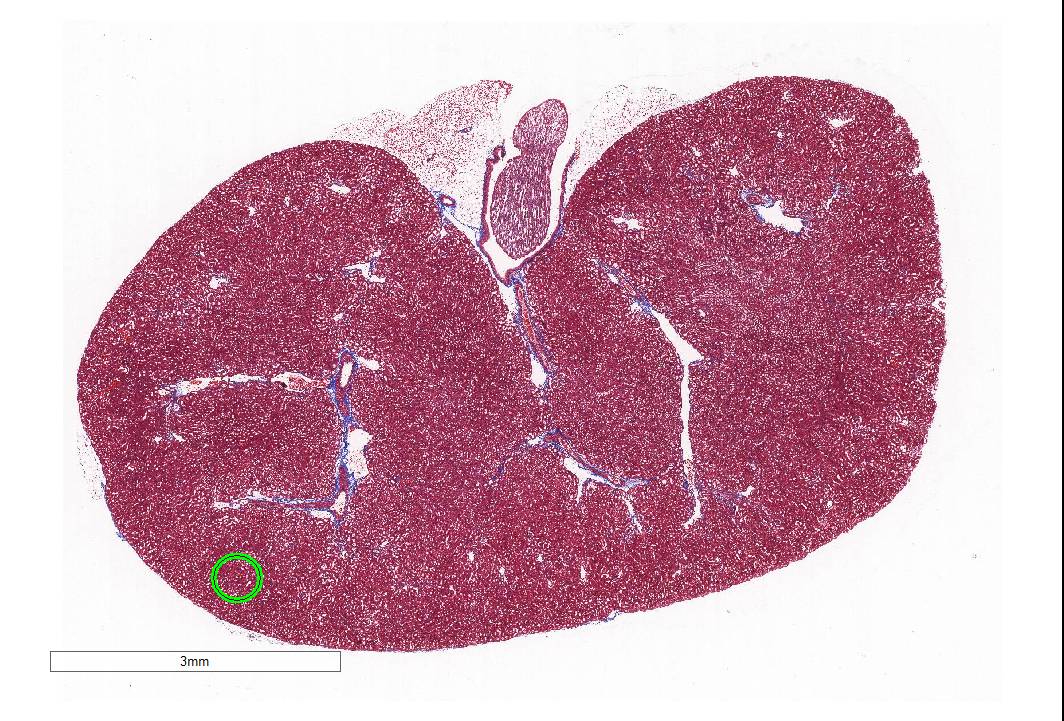

Supplement: MASSON C group.jpg [file IRNF_A_2561221_SM0240.jpg]

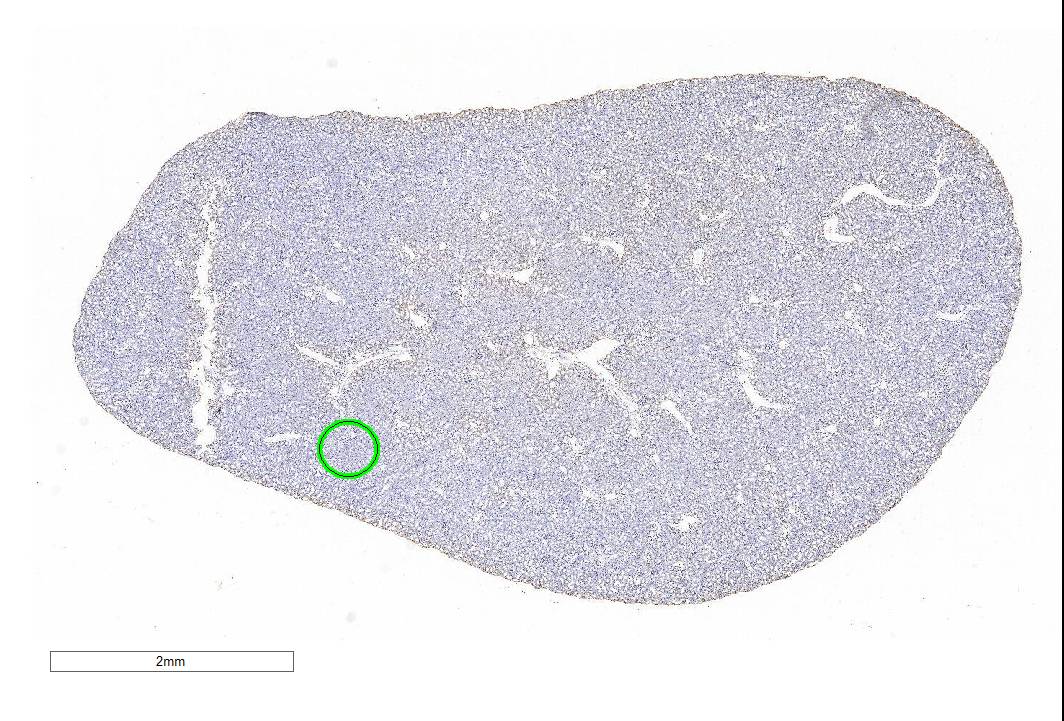

Supplement: TUNEL HE group.jpg [file IRNF_A_2561221_SM0239.jpg]

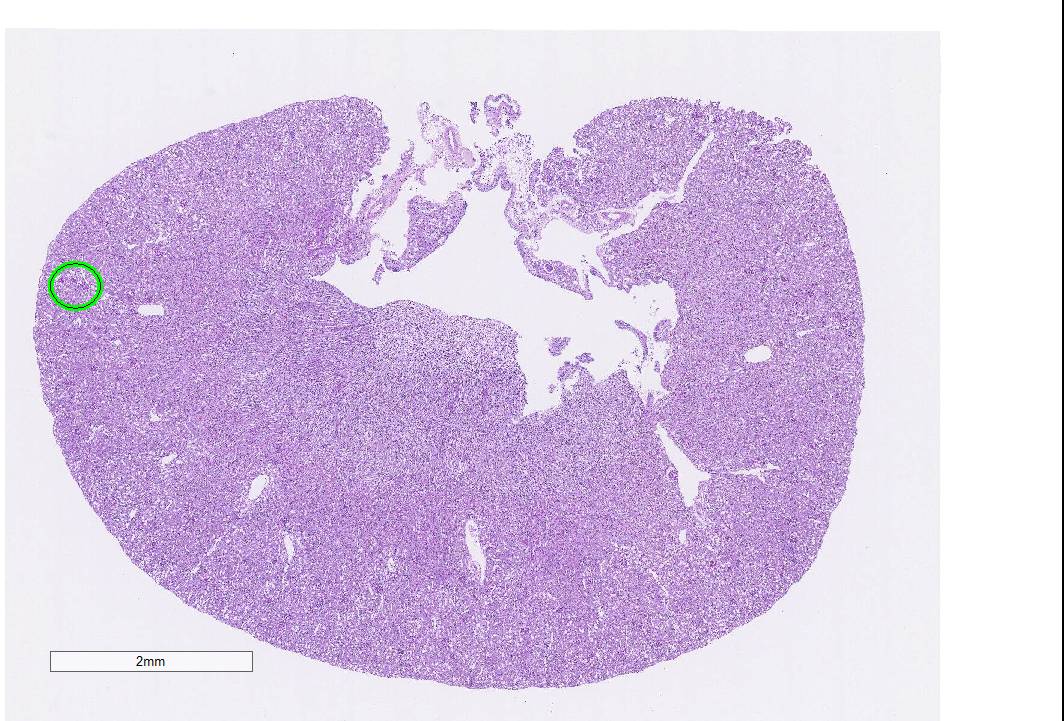

Supplement: PAS H group.jpg [file IRNF_A_2561221_SM0238.jpg]

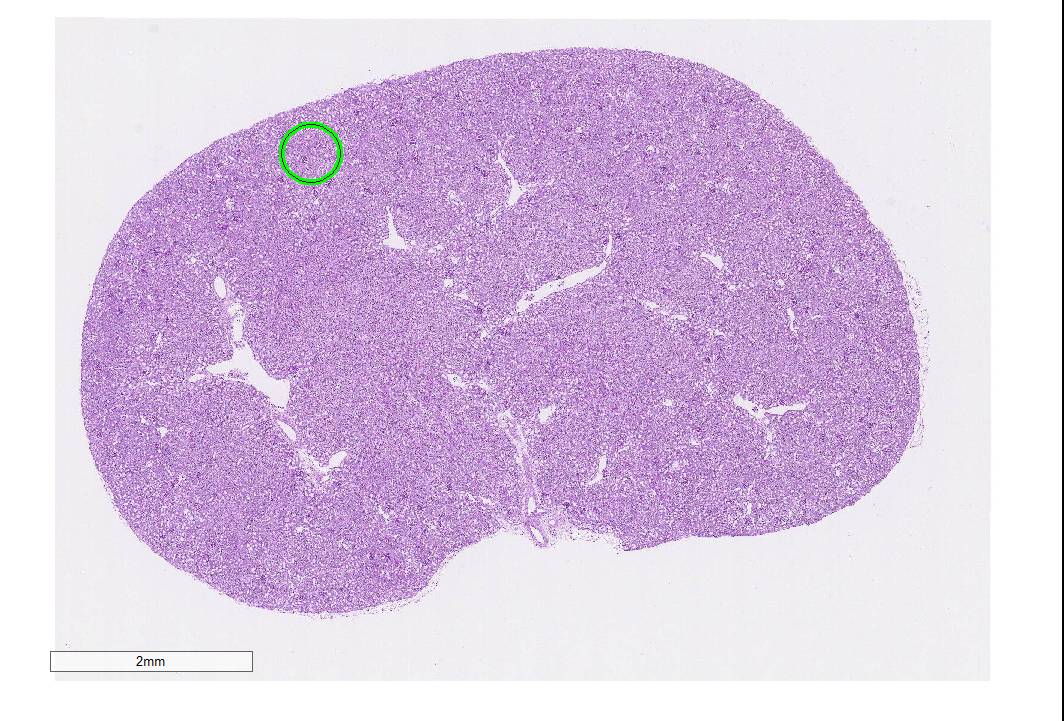

Supplement: PAS C group.jpg [file IRNF_A_2561221_SM0237.jpg]
